# The Role of Antegrade Irrigation via Percutaneous Nephrostomy on Surgical Outcomes in Semirigid Ureteroscopy among Patients with Upper Ureteral Stones

**DOI:** 10.1155/2019/8657609

**Published:** 2019-07-04

**Authors:** Wonho Jung, Hye Jin Byun, Dong Sup Lee

**Affiliations:** ^1^Department of Urology, Dongsan Medical Center, Keimyung University School of Medicine, Daegu, Republic of Korea; ^2^School of Medicine and Institute for Medical Science, Keimyung University, Dongsan Medical Center, Daegu, Republic of Korea; ^3^Department of Urology, St. Vincent's Hospital, The Catholic University of Korea, Suwon, Republic of Korea

## Abstract

**Objective:**

We aimed to investigate the role of antegrade irrigation via percutaneous nephrostomy on surgical outcomes in retrograde ureteroscopy in patients with upper ureter stones.

**Materials and Methods:**

In this retrospective study, we analyzed 134 patients who underwent retrograde semirigid ureteroscopy for upper ureter stones between August 2012 and December 2017. Patients were divided into two groups: retrograde irrigation group (conventional URS) and antegrade irrigation group (using percutaneous nephrostomy). Operation time, postoperative hospital stay, complications, and stone-free rate were measured for each patient after ureteroscopy.

**Results:**

The mean age in the retrograde irrigation and antegrade irrigation groups was 53.3 and 60.7 years, respectively (p=0.007). The operation time was 60.8 min vs. 43.0 min (p=0.002), and stone-free rate was 82.0 % vs. 95.5 % (p=0.033). Stone size, laterality, the proportion of male patients, and urinary tract infection prevalence were comparable between the groups. In the subgroup analysis of stone size >10 mm, the antegrade irrigation group had a shorter operation time and a higher stone-free rate. For stone size of 5–10 mm, operation time in the antegrade irrigation group was shorter and the stone-free rate between the two groups was comparable.

**Conclusion:**

Antegrade irrigation via percutaneous nephrostomy during ureteroscopy has a higher stone-free rate with a shorter operation time without an increased urinary tract infection risk. Therefore, if percutaneous nephrostomy is necessary before ureteroscopy, antegrade irrigation of external fluid via percutaneous nephrostomy is strongly recommended.

## 1. Introduction

According to a report, over 70% of patients with ureter stones experienced severe pain (visual analog scale 7 or more) [1]. In the United States, over one million patients with urolithiasis visit the emergency room per year [2]. Thus, the primary goal of treatment in the emergency department for renal colic due to urolithiasis is pain relief; whenever possible, a nonsteroidal anti-inflammatory drug (NSAID), such as diclofenac, indomethacin, or ibuprofen, is the first choice [3]. However, urinary diversion, such as percutaneous nephrostomy (PCN) or ureteral stenting, may be required in patients with ureter stones complaining of intractable pain despite treatment with analgesics. Moreover, especially in elderly patients and patients with hypertension, transient renal impairment could be worsened by repeated NSAID administration [4]. For urinary diversion, PCN may be preferred for hydronephrosis due to urinary stones [5].

Active treatment for upper ureter stone could be the most challenging for urologists. Semirigid ureteroscopy, which is the most commonly performed procedure in the management of ureter stones [6], has shown poor results with a stone-free rate (SFR) of approximately 80% for upper ureter stone, which could be mainly attributed to retropulsion (upward migration) of the stone; consequently, additional procedure(s) such as extracorporeal shock wave lithotripsy (ESWL) is needed [7, 8]. Hence, a guideline recommended percutaneous antegrade ureteroscopy when retrograde ureteroscopy for upper ureter stone fails [9]. Nonetheless, flexible ureteroscopy could be the best option for upper ureter stones [10] as it could substantially improve SFR (90–100%) and greatly decrease complication rates [8]. However, its durability is a major concern [11]. Furthermore, given the high cost and maintenance, the cost-benefit ratio is a significant factor when considering flexible ureteroscopy, especially in developing countries [12, 13].

The irrigation flow from the kidney to the bladder may get rid of the fear of ‘retropulsion.' Therefore, if percutaneous nephrostomy (PCN) has been performed before URS, the antegrade irrigation via PCN may increase stone-free rate and reduce the ancillary procedures, such as flexible ureteroscopy or percutaneous antegrade ureteroscopy. Nevertheless, to the best of our knowledge, only one clinical research where PCN was utilized during retrograde semirigid ureteroscopy (the irrigation fluid flowed via the PCN tube and not via the ureteroscope) was conducted [14]. It may be due to the facts that (1) PCN cases in the upper ureter stones are not common because PCN is generally considered in patients with intractable pain, suspicious infection, or deteriorating renal function; (2) surgeons may be concerned that the antegrade irrigation via PCN during URS may increase the infection rate. Thus, this study aimed to investigate antegrade irrigation via PCN in retrograde ureteroscopy. We compared the surgical outcomes of antegrade irrigation method with PCN with those of retrograde irrigation method (conventional URS) during ureteroscopy for upper ureter stone.

## 2. Materials and Methods

### 2.1. Patients and Protocols

We retrospectively analyzed 134 patients who underwent semirigid ureteroscopy for upper ureteral stones (stone size, >5 mm) between August 2012 and December 2017. The subjects were divided into two groups, depending on the flow of irrigation during surgery: antegrade flow group via PCN (n=45) and retrograde flow group via ureteroscope (n=89). To evaluate operative outcomes, we compared age (years), body mass index, sex, stone size, operation time, presence of urinary tract infection (UTI), SFR, and complication rates. All PCN procedures (8.5 Fr. Dawson Mueller Drain, Cook, Indianapolis, USA) were performed under ultrasonographic guidance with local anesthesia in patients with (1) severe pain that did not subside within 1 day despite repeated NSAID administration or (2) worsening renal function. After the operation, the PCN catheter was removed under fluoroscopy and a double-J ureteral stent was placed for 7 days.

**Figure 1:**
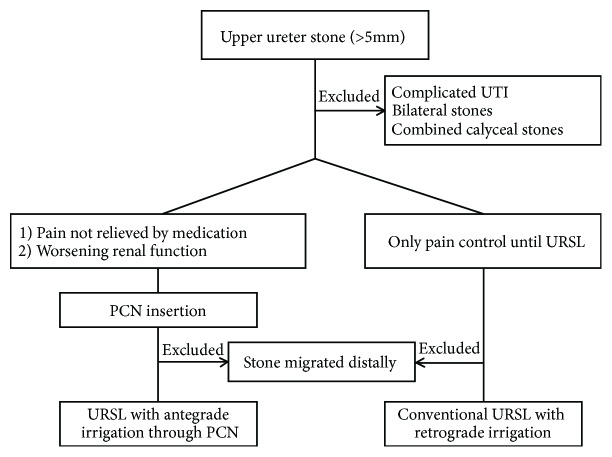
Study paradigm. When percutaneous nephrostomy was inevitable in patients with upper ureter stone, antegrade irrigation of external fluid via percutaneous nephrostomy was performed and its outcomes were compared with those of conventional semirigid ureteroscopy. URSL, ureteroscopic lithotripsy; PCN, percutaneous nephrostomy; UTI, urinary tract infection.

Complications were evaluated according to the modified Clavien grading system [15]. Postoperative febrile UTI was defined as body temperature >38°C without any symptoms except those related to the urinary tract and was classified as grade I (febrile UTI without additional treatment), grade II (febrile UTI with additional antibiotic treatment), grade III (sepsis without intensive care unit management), or grade IV (sepsis with intensive care unit management). In addition, stone-free status was defined as no obvious stones based on nonenhanced abdominal computerized tomography at 1 month after ureteroscopy. Exclusion criteria included the following: preoperative systemic infection (fever >38°C), calyceal stone, bilateral stone, and lower/midureter stone (Figure 1).

### 2.2. Perioperative Procedures

#### 2.2.1. Semirigid Ureteroscopy

In this study, ureteroscopy was performed with patients in the lithotomy position under spinal or general anesthesia using a 6.5/7 Fr semirigid ureteroscope (Karl Storz Endoscope, Tuttlingen, Germany). Holmium-YAG laser lithotripter (VersaPulse PowerSuite, Lumenis Surgical, San Jose, CA, USA) was used for stone fragmentation and a 365-*μ*m laser fiber was employed (power setting: 0.8–1.0 J × 10–15 Hz).

#### 2.2.2. Antegrade Irrigation and Renal Pelvis Pressure Measurement

**Figure 2:**
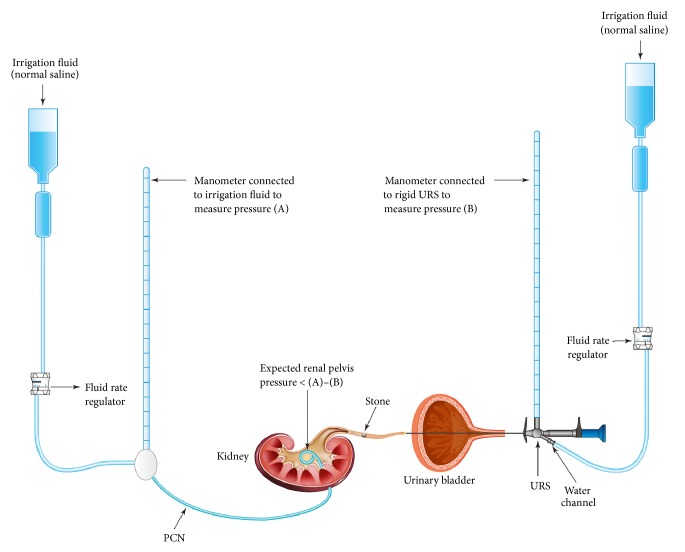
Pressure drop (renal pelvis pressure) measurement during ureteroscopic lithotripsy with antegrade irrigation. Once the stone was fragmented, antegrade irrigation was initiated via percutaneous nephrostomy tube while simultaneously removing the retrograde irrigation line from a water channel of the ureteroscope. At this time, pressure drop could be measured by subtracting (B) from (A). PCN, percutaneous nephrostomy; URS, ureteroscope.

In patients with antegrade irrigation via PCN, the renal pelvis pressure was measured. The irrigation fluid was connected to a manometer and a PCN tube using a three-way connector. Generally, the antegrade irrigation pressure was set to slightly higher than 40 cmH_2_O. A ureteroscope was introduced into the ureter, and one fluid-connecting channel of the ureteroscope was attached to another irrigation fluid (similar to that in retrograde irrigation). In cases of an impacted stone, retrograde irrigation was initially performed until the impacted stone cracked or until the stone was detached from ureteral wall. Subsequently, the stone crack or peristone space enabled the antegrade irrigation to flow. The antegrade flow was facilitated by a safety guidewire (Supplementary Material (available here)). When the antegrade irrigation fluid flowed downward through the stone, we disconnected the retrograde irrigation channel and opened the water channel of the ureteroscope. During ureteroscopy with antegrade irrigation alone, we measured the pressure of obstructed lesion by connecting a manometer to the opened water channel of the ureteroscope. Subsequently, pressure drop was measured (Figure 2).

### 2.3. Statistical Analysis

Student's* t*-test was used to compare the mean of continuous variables and Mann-Whitney* U* test to compare interval scales. Binominal variables were compared using chi-square test or Fisher's exact test. A* p* value <0.05 was considered statistically significant for all tests. Analyses were conducted using the SPSS ver. 25.0 (SPSS Inc., Chicago, IL, USA).

## 3. Results

### 3.1. Characteristics of the Subjects

Patients in the antegrade irrigation group were significantly older (60.7 vs. 53.3 years, p = 0.007). Other baseline characteristics were not different between the two groups (Table 1).

### 3.2. Surgical Outcomes between Antegrade Irrigation and Retrograde Irrigation

The antegrade irrigation group showed significantly better outcomes in SFR (antegrade vs. retrograde, 95.6 % vs. 82.0 %, respectively; p = 0.033) and operation times (antegrade vs. retrograde, 42.9 vs. 60.8 min; p = 0.002) (Table 2). In the subgroup analysis (Table 3), the antegrade irrigation group showed a shorter operation time regardless of stone size. However, the SFR was not different between the two groups when the stone size was <10 mm. Moreover, postoperative hospital stay and the incidence of procedure-related UTI were comparable between the two groups. Mean±standard deviation of the pressure drop in the antegrade irrigation group was 9.17±3.17 cmH2O.

### 3.3. Predictive Factors for Postoperative UTI

Antegrade irrigation did not influence the occurrence of UTI. Preoperative bacteriuria (p = 0.001), nitrituria (p = 0.019), and pyuria (p = 0.025) were more significant factors for UTI than operation time or age (Table 4).

## 4. Discussion

In this study, we compared the surgical outcomes between antegrade irrigation with PCN and retrograde irrigation without PCN during ureteroscopy. Antegrade irrigation is associated with higher SFR and lower operation times compared with retrograde irrigation, which indicates that surgical outcomes in patients with upper ureter stone with preoperative PCN are better than those without.

For upper ureteral stones, stone retropulsion can occur in >20 % of ureteroscopy cases [16], which results in increased operation times and secondary procedures due to residual stones. Thus, various antiretropulsion devices are being used during ureteroscopy for upper ureteral stones. A meta-analysis on the use of N Trap showed a significant increase in SFR (OR=3.08) and decreased incidence of stone retropulsion (OR=0.23). However, no significant difference in operation time was found [17]. Sun et al. suggested an ingenious method to prevent stone migration into the renal pelvis during ureteroscopy; the tip of a 4 Fr ureteral catheter was placed beyond the upper ureter stone, which was followed by irrigation through the catheter [18]. Their approach is quite similar to that of our study.

Generally, PCN could be employed for relief of urinary obstruction, which could be due to (1) suspected infection, (2) worsening of renal function (acute renal failure), and (3) intractable pain [19]. The choice between PCN and double-J stent placement can be influenced by multiple factors including physician's specialty at initial clinical presentation, disease severity, stone size, location of stone, modality of definitive stone management, or availability of in-hospital interventional radiology services [20]. In Korea, when patients visit the department of emergency due to acute pain caused by ureter stones which is nonresponding to medications and when emergent operation such as ureteroscopy is unavailable, it is common that interventional radiologists support the preoperative pain management by placement of PCN, which has little burden on the use of the operating room and anesthesia. Moreover, double-J stenting using rigid cystoscopy by local anesthesia is painful, especially in male patients [21, 22]. In addition, when ureter stone(s) is large and/or multiple, there is a possibility of ureteral stenting failure. Pandey et al. experienced 13.9% of ureteral stenting failure in obstructive stone management [23]. Thus, in cases of ureter stones with intractable pain, we prefer PCN placement rather than double-J stenting. In this study, only PCN was collected to evaluate the effect of antegrade and retrograde irrigation. There was no complication associated with PCN application in this study.

In our study, to compare the outcomes between the antegrade and retrograde irrigation groups, patients who had to undergo PCN insertion because of ureter stone with suspected infection were excluded. A previous study used antegrade irrigation during retrograde ureteroscopy, which is a method similar to ours; 42 patients had PCN and antegrade irrigation was applied in 21 patients [14]. They found that, with antegrade irrigation method during retrograde ureteroscopy, fragmented stones could be more easily shifted from the proximal to the distal ureter. In our study, we observed that antegrade irrigation during ureteroscopy could reduce the need for an instrument, such as a basket, and the stone was removed from the upper ureter to the bladder without any risk of ureter avulsion [24], thereby resulting in a shortened operation time. Moreover, in the subgroup analysis of stone size >10 mm, the anterograde irrigation group had a shorter operation time and a higher SFR than the retrograde irrigation group. For cases with stone size <10 mm, only a shorter operation time was noted. Therefore, if PCN is inevitable, antegrade external fluid irrigation during retrograde semirigid ureteroscopy is highly recommended. It could not only produce a clear visual field but also result in an extremely low stone migration rate, and it is beneficial because the related cost and morbidity of the ancillary procedures could be reduced [25].

According to several studies, the prevalence rate of urinary sepsis after ureteroscopy was 4~8% [26, 27]. Even in the patients with negative preoperative urine culture, UTI or urinary sepsis could be developed after ureteroscopy although the prevalence rate was reported lower than 2.2% [28]. In this study, antegrade irrigation was performed with gravity instead of positive pressure. Intrarenal pressure is a major factor in postoperative morbidity, including infection [14, 29, 30]. However, our study showed no difference in UTI prevalence between the two groups, which could be because the intrarenal pressure possibly passed from the renal pelvis to an open water channel of the ureteroscope. Moreover, for impacted stone, which interrupts the irrigation flow, retrograde irrigation may be a better initial approach until the impacted stone is fragmented. Subsequently, antegrade irrigation could be initiated while simultaneously removing the retrograde irrigation line from a water channel of the ureteroscope (Figure 2 and Supplementary Material). In addition, as previously described, a possible increased renal pelvis pressure is a major concern in antegrade irrigation during retrograde ureteroscopy. To understand renal pelvis pressure during ureteroscopy with antegrade irrigation, it is helpful to recall the Whitaker test. Whitaker developed a method to measure the pressure between the renal pelvis and the bladder for differentiating urinary tract obstruction [31]. It was postulated that a pressure drop across the site of suspected obstruction, which is obtained by subtracting the bladder pressure from the absolute pressure (pressure within the renal pelvis produced by perfusion of the nephrostomy), of <15 cmH2O means no obstruction and a pressure drop of >22 cmH2O indicates obstruction. In our study, during antegrade irrigation (i.e., after stone fragmentation when fluid could flow distally, passing through the stone), we set the absolute pressure to slightly higher than 40 cmH_2_O. The mean±standard deviation of the pressure drop was 9.17±3.17 cmH2O with antegrade irrigation. However, this measurement value could be overestimated because of the following: (1) when the stone fragments jammed in the ureter, the measured value was high; when the fragments were floating freely in the ureter, the value was low; (2) the renal pelvis and ureter are not a rigid pipeline (especially because of hydronephrosis); and (3) a small fraction of the absolute pressure could pass through the periureteroscopic space.

Furthermore, antegrade irrigation during ureteroscopy in this study exerted no influence on UTI development postoperatively. This finding differs from that of a previous study [14], which could be attributed to the methods employed in our study (i.e., antegrade irrigation was started at the time of stone fragmentation and the irrigation was not squeezed but was allowed to naturally drain). Moreover, age, operation time, and underlying diabetes mellitus were not causative factors of UTI in our study, whereas preoperative bacteriuria, nitrituria, and pyuria were identified as predisposing factors for UTI, as previously suggested [32]. In addition, despite the routine prophylactic antibiotic administration, UTI developed in eight of 11 patients with pyuria, in three of 11 with nitrituria, and in seven of 11 with asymptomatic bacteriuria. Prior to urological surgery, treatment for asymptomatic bacteriuria is recommended [33]. However, for afebrile patients with pyuria, nitrituria, or bacteriuria, the duration for prophylactic antibiotic administration before ureteroscopy has not been established. Thus, urine culture should be performed when urinalysis before ureteroscopy showed abnormal results; it could help surgeons decide whether to maintain the previous prophylactic antibiotic or to change it especially when UTI develops after ureteroscopy.

The greatest limitation of our study is its retrospective nature and relatively small sample size. Thus, a well-designed prospective study with a large population is warranted to further validate our findings. Despite the limitations, our study showed several advantages of antegrade irrigation during ureteroscopy in patients with upper ureter stones.

## 5. Conclusions

Ureteroscopy of the proximal ureter using the antegrade irrigation method through PCN resulted in a higher SFR and a shorter operation time without an increase in complication rate. If PCN is inevitable before ureteroscopy, anterograde irrigation for the management of proximal ureter stones is strongly recommended to prevent stone retropulsion and reduce additional procedures.

## Figures and Tables

**Table 1:** Baseline characteristics.

	Retrograde (n=89)	Antegrade (n=45)	*p*-value
Age (years)	53.33 ± 14.60	60.73 ± 15.00	0.007^a^
Body mass index (kg/m^2^)	25.89 ± 3.45	24.84 ± 3.63	0.104^a^
Stone size (maximal)	9.34 ± 3.51	10.23 ± 4.34	0.204^a^
Hounsfield unit	703.93 ± 302.60	623.85 ± 234.15	0.124^a^
Sex (female/male)	33/56	22/23	0.199^b^
Laterality (Rt/Lt)	39/50	18/27	0.714^b^
Diabetes mellitus (yes/no)	26/63	15/30	0.693^b^
Hypertension (yes/no)	39/50	24/21	0.360^b^
Previous ESWL (yes/no)	26/63	12/33	0.841^b^
Main stone component^c^			
Calcium oxalate	49/67	20/25	0.785^b^
Uric acid	8/67	4/25	0.330^d^
Struvite	3/67	1/25	
Others	7/67	0/25	

Data were expressed as mean ± standard deviation or number of relevant cases.

ESWL: extracorporeal shock wave lithotripsy.

a: Student's *t*-test; b: chi-square test; d: Fisher's exact test.

c: stone analyses were done in 92 cases (68.7%) of 134 patients (67 in retrograde group and 25 in antegrade group).

**Table 2:** Main surgical outcomes.

	Retrograde (n=89)	Antegrade (n=45)	*p* value
Operative time (min)	60.82 ± 32.29	42.96 ± 26.99	0.002^*∗*^
Postoperative hospital stay (days)	1.67 ± 1.78	2.87 ± 4.65	0.299^*∗∗*^
Stone-free (yes/no)	73/16	43/2	0.033^*∗∗∗*^
UTI (yes/no)	5/84	5/40	0.303^*∗∗∗∗*^
Steinstrasse (yes/no)	3/86	0/45	NA

Data were expressed as mean ± standard deviation or number of relevant cases.

UTI: urinary tract infection.

*∗*Student's *t*-test.

*∗∗*Mann-Whitney *U* test.

*∗∗∗*Chi-square test.

*∗∗∗∗*Fisher's exact test.

**Table 3:** Subgroup analysis according to stone size.

Stone size >10 mm	Retrograde (n=24)	Antegrade (n=20)	*p* value
Operation time (min)	87.96 ± 34.91	54.05 ± 31.17	0.002^*∗*^
Postoperative hospital stay (days)	1.79 ± 1.06	3.80 ± 5.45	0.526^*∗∗*^
Stone-free (yes/no)	14/10	18/2	0.039^*∗∗∗*^
UTI (yes/no)	2/22	3/17	0.646^*∗∗∗∗*^

Stone size 5–10 mm	Retrograde (n=65)	Antegrade (n=25)	*p* value

Operation time (min)	50.80 ± 24.86	34.08 ± 19.53	0.003^*∗*^
Postoperative hospital stay (days)	1.63 ± 1.98	2.12 ± 3.84	0.694^*∗∗*^
Stone-free (yes/no)	59/6	25/0	0.181^*∗∗∗*^
UTI (yes/no)	3/62	2/23	0.615^*∗∗∗*^

Data were expressed as mean ± standard deviation or number of relevant cases.

UTI: urinary tract infection.

*∗*Student's *t*-test.

*∗∗*Mann-Whitney *U* test.

*∗∗∗*Chi-square test.

*∗∗∗∗*Fisher's exact test.

**Table 4:** UTI risk evaluation with pre- and perioperative data.

	With UTI	With UTI	*p* value
	Yes	No	(OR, 95 % confidence interval)
Antegrade irrigation	5/45	6/89	0.506^*∗*^ (1.72, 0.498–6.007)
Preoperative pyuria	8/53	3/81	0.025^a*∗*^ (4.62, 1.167–18.312)
Preoperative nitrituria	3/8	8/126	0.019^*∗*^ (8.85, 1.789–48.855)
Perioperative bacteriuria	7/24	4/110	0.001^*∗*^ (10.91, 2.883–41.296)
Sex (male)	8/79	3/55	0.524^*∗*^ (1.95, 0.494–7.719)
Underlying DM	1/41	10/93	0.172^*∗*^ (0.208, 0.026–1.678)
Previous ESWL	4/38	7/96	0.506^*∗*^ (1.496, 0.412–5.436)

	UTI +	UTI –	*p* value
	(n=11)	(n=123)	(95 % confidence interval)

Age (years)	58.45 ± 15.61	55.57 ± 15.09	0.547^*∗∗*^ (-12.296–6.542)
Operation time (min)	55.91 ± 30.07	54.72 ± 31.92	0.903^*∗∗*^ (-21.890–19.518)

UTI, urinary tract infection; OR, odds ratio; DM, diabetes mellitus; ESWL, extracorporeal shockwave lithotripsy.

*∗*Fisher's exact test.

*∗∗*Student's *t*-test.

## Data Availability

The Excel data used to support the findings of this study have been deposited in the ‘Open Science Framework' repository.

## Supplementary Materials

Supplementary Materials This video shows ureteroscopic ureterolithotripsy for upper ureter stones (>10 mm, Hounsfield unit 790). Continuous antegrade irrigation fluid from the kidney to the ureteroscope results in a clear visual field during laser ureteroscopic treatment. Furthermore, antegrade irrigation flow not only prevents stone retropulsion but also facilitates spontaneous passage of the stone fragments without basketing.
